# Self-assembly of stabilized droplets from liquid–liquid phase separation for higher-order structures and functions

**DOI:** 10.1038/s42004-024-01168-5

**Published:** 2024-04-09

**Authors:** Mehwish Naz, Lin Zhang, Chong Chen, Shuo Yang, Hongjing Dou, Stephen Mann, Jianwei Li

**Affiliations:** 1 State Key Laboratory of Metal Matrix Composites, School of Materials Science and Engineering, Shanghai Jiao Tong University, Shanghai, 200240 China; 2 Zhangjiang Institute for Advanced Study (ZIAS), Shanghai Jiao Tong University, 429 Zhangheng Road, Shanghai, 201203 China; 3 MediCity Research Laboratory, University of Turku, Tykistökatu 6, Turku, 20520 Finland; 4 Centre for Protolife Research and Centre for Organized Matter Chemistry, School of Chemistry, University of Bristol, Bristol, UK; 5 Max Planck-Bristol Centre for Minimal Biology, School of Chemistry, University of Bristol, Bristol, BS8 1TS UK

**Keywords:** Supramolecular chemistry, Self-assembly

## Abstract

Dynamic microscale droplets produced by liquid–liquid phase separation (LLPS) have emerged as appealing biomaterials due to their remarkable features. However, the instability of droplets limits the construction of population-level structures with collective behaviors. Here we first provide a brief background of droplets in the context of materials properties. Subsequently, we discuss current strategies for stabilizing droplets including physical separation and chemical modulation. We also discuss the recent development of LLPS droplets for various applications such as synthetic cells and biomedical materials. Finally, we give insights on how stabilized droplets can self-assemble into higher-order structures displaying coordinated functions to fully exploit their potentials in bottom-up synthetic biology and biomedical applications.

## Abstract (web summary)

Dynamic microscale droplets produced by liquid–liquid phase separation (LLPS) have emerged as appealing biomaterials, but their instability hinders their assembly into high-order structures with collective behaviors. Here, the authors review current strategies for stabilizing droplets, as well as recent developments in the applications of such LLPS droplets, and provide insights into how stabilized droplets can self-assemble into higher-order structures that display coordinated functions.

## Introduction

Liquid-liquid phase separation (LLPS), a phenomenon where a homogenous liquid solution demixes into two distinct phases, is determined by the intricate interplay between intermolecular interactions and external environment. At the core of this phenomenon is the nucleation, growth and dynamics of membrane-less molecularly crowded liquid droplets. LLPS droplets are observed frequently in living cells where they exhibit diverse functions ranging from signaling pathways to metabolic processes^1^. Inspired by their profound biological implications, LLPS droplets have increasingly garnered attention in bottom-up synthetic biology and bioengineering as agents for constructing hierarchically structured materials with life-like collective behaviors. Yet, the very nature of LLPS droplets, characterized by their dynamic, fluid-like properties, comes with an intrinsic instability^2^. Therefore, various stabilization strategies have been developed to address this challenge.

This review presents an overview of dynamic droplets resulting from LLPS and their applications in artificial cells and biotechnological settings. We begin by discussing the formation and materials properties of liquid droplets. Next, we investigate the inherent instability of LLPS droplets and current stabilization strategies, such as physical separation and chemical modulation. The exploitation of LLPS properties and structures to confer biomimetic behaviors and biochemical applications are then discussed. Finally, the review concludes with a perspective on how stabilized LLPS droplets can be harnessed to assemble into multicompartment complex structures with collective behaviors, unleashing their diverse potential.

### Formation of LLPS droplets

**Fig. 1:**
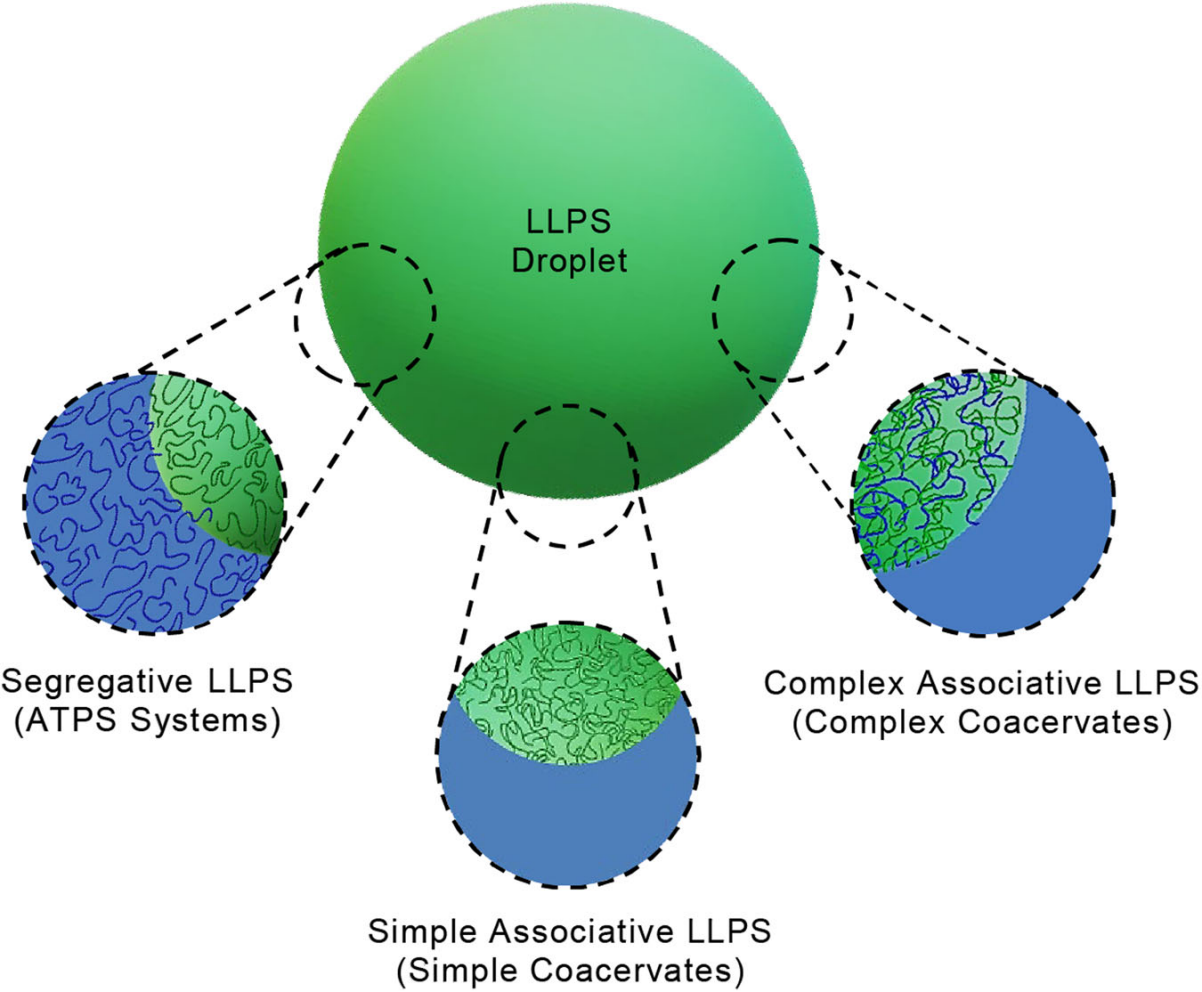
Liquid-liquid phase separation (LLPS) droplet formation. A schematic overview illustrating different strategies of LLPS droplet formation: segregative, complex and simple associative LLPS.

LLPS can be broadly categorized into three modes of formation: segregative, associative, and simple phase separation (Fig. 1). In segregative LLPS, the primary driving force arises from the mutual repulsion between different (macro)molecules in a solution. A common system demonstrating this phenomenon is the combination of polyethylene glycol (PEG) and dextran. These two polymers, propelled by their mutual incompatibility, segregate into distinct aqueous two-phase system (ATPS): one phase enriched with PEG and the other predominantly with dextran^3^. Other common examples of ATPS involve single polymers and salts^4^. In the case of associative LLPS, more commonly termed as “coacervation,” different (macro)molecules generate attractive interactions that facilitate their complexation, resulting in phase-separated systems (complex coacervates)^5^ comprising a solute-rich phase (the coacervate phase) and a solute-depleted phase (the supernatant phase). Attractive interactions can be driven by a variety of molecular forces including nonspecific interactions^6,7^ such as charge-based interactions, hydrogen bonding, hydrophobic interactions and π-π interactions and specific interactions including nucleic acid base pairing^8^ and protein recognition^9^. The basic form of coacervation consists of two polyelectrolytes with opposite charges^5,6^, while biomolecule-based coacervation usually involves a combination of nonspecific and specific interactions^10^. Simple phase separation occurs when a single type of solute self-associates through intermolecular attractive interactions. The chemical stability of LLPS systems, encompassing both ATPS and coacervate systems, is influenced by factors including pH, ionic strength, and temperature, which impact their intermolecular interactions^10–12^. Recently, segregative and associative interactions have been combined to form complex droplets with functions that have not been realized for either ATPS or coacervation^13,14^.

In conclusion, both segregative and associative LLPS systems exhibit dynamical properties that are innately endowed with the capability to adapt and evolve in reaction to surrounding changes, ensuring their functionality under various conditions.

## Mechanical stability of LLPS droplets

After formation, the mechanical properties of LLPS droplets play a crucial role in determining their behaviors. LLPS droplets have unique mechanical properties including visco-elasticity and interfacial tension that are influenced by molecular characteristics and external environmental conditions. Understanding these aspects is essential for controlling their behaviors for various applications. For example, the viscosity of the PEG/dextran ATPS system increases with polymer concentrations and molecular weights to give a limited range of 10–100 Pa·s^15^. On the other hand, polyelectrolyte-based coacervates cover a wider range (approximately 0.1–1000 Pa·s)^5,16^, contingent on the chemical structures and lengths of the polyelectrolytes, as well as external parameters such as salt concentrations and temperature^17,18^. In addition, the biomolecule-based coacervates have a broader range of complex interactions compared to polyelectrolyte-based ones, potentially leading to comparatively higher viscosity than polyelectrolyte-based coacervates^5,19,20^.

Another mechanical property that influences the stability of the droplets is interfacial tension, which is the force per unit length acting on the interface of two immiscible phases. Segregative and associative phase separations exhibit distinctive interfacial tension. The PEG/dextran system has an interfacial tension of about 1–100 μN·m^−1^ ^15^. Influential factors include stoichiometry, molecular weights of the polymers, salt concentrations and temperature. On the other hand, coacervate droplets have a higher interfacial tension, typically between 10 and 500 μN·m^−1^ ^5^. Collectively, LLPS droplets generated from different components have unique viscosities and interfacial properties which could have important implications on their feasibility in various applications.

**Fig. 2:**
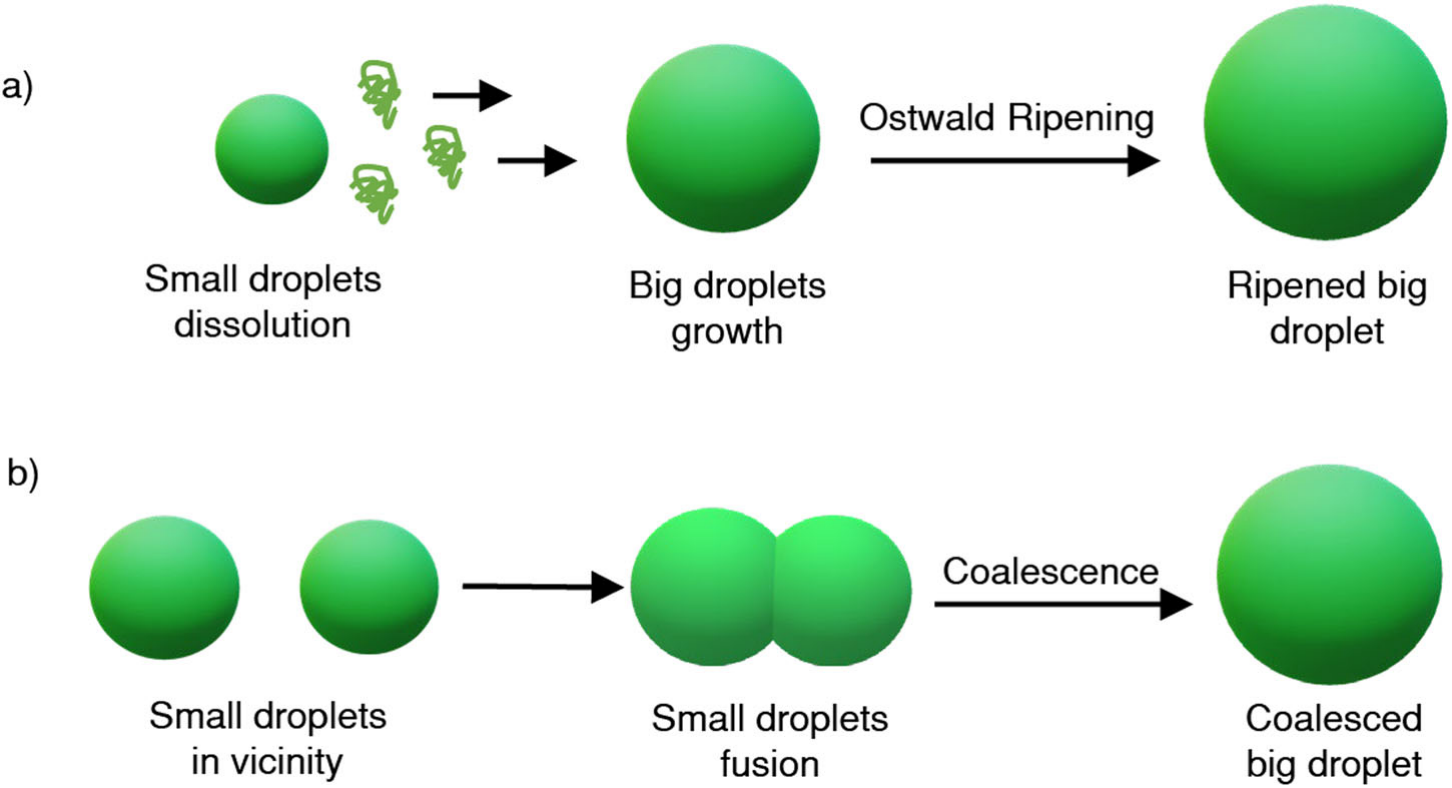
Schematic illustration of droplet instability due to Ostwald ripening and coalescence. **a** Ostwald ripening: Large droplets grow at the expense of small unstable droplets. Over time, larger ripened droplets with reduced surface tension and area are observed. **b** Coalescence: Small droplets coalesce to minimize the surface tension. Over time, coalesced large droplets in a comparatively stable conformation with reduced surface tension and surface area are formed.

The unique mechanical properties of LLPS droplets lead to the inherent unstability of the LLPS droplets, which will eventually phase separate macroscopically via Ostwald ripening and coalescence^21^ (Fig. 2). Ostwald ripening is a process in which larger droplets develop at the expense of smaller ones due to the differences in solubility or vapor pressure in solutions or colloidal suspensions. On the other hand, coalescence is the process by which two or more droplets merge to produce a bigger droplet, facilitated by surface tension which promotes phase separation by reducing the energy barrier for the formation of new interfaces between the phases^22^. In ATPS systems, higher surface tensions can accelerate this process, as smaller droplets with higher chemical potential are prone to disassemble. Factors such as polymer concentrations and molecular weights influence the ripening behavior^23^. For coacervates, surface charges and differences in osmotic pressure between droplets play a pivotal role^24^. In the PEG/Dextran system, droplets with high surface tension might resist merging, maintaining their individual identities longer. The intrinsic interactions between the polymers, as well as factors such as concentration and molecular weight, can modulate this behavior^25,26^. Alternatively, the net surface charge of coacervate droplets can control coalescence: non-neutral surface charges prevent coalescence by electrostatic repulsion. Additionally, the surrounding medium viscosity can influence the rate of droplet movement and collision, impacting coalescence^27^. Collectively, LLPS droplets tend to grow passively with decreased number of droplets due to thermodynamically instability, which requires stabilized strategies for long term use in applications.

## LLPS droplet stabilization strategies

Next, we describe methods used for stabilizing LLPS droplets, which mainly fall into two approaches: physical separation and chemical modulation. Physical separation methods involve using external forces, hydrogel matrices and membranization to stabilize the LLPS droplets as well as control the droplet dynamics (Table 1).

**Table 1:** Overview of examples of LLPS droplet stabilization strategies

LLPS droplet stabilization strategies	Methods	Refs.
Physical Separation	External Forces	Acoustic Field^28^, Electric Field^32^
	Hydrogel Matrices	Agarose Hydrogel^33^
	Membranization	Fatty Acid^36^, Lipid^37^, Metal-Phenolic Network^38^, Protein^39,135^, Polymer^42,45^, Polyelectrolytes^40,41^
	Nano/Micro-Objects	Nanowires^46,47^, Nanoplates^48^, Nano-Microgels^49^, Nanorods^50^, Vesicles^51^
	Living Systems	Cell Fragments^52,53^/Living Cells^54^
Chemical Modulation	Oxidative Crosslinking	Tyrosine^58^
	Non-Covalent Interactions	DNA^60^
	Chemical Fuelling	ATP^21^

### Physical separation

**Fig. 3:**
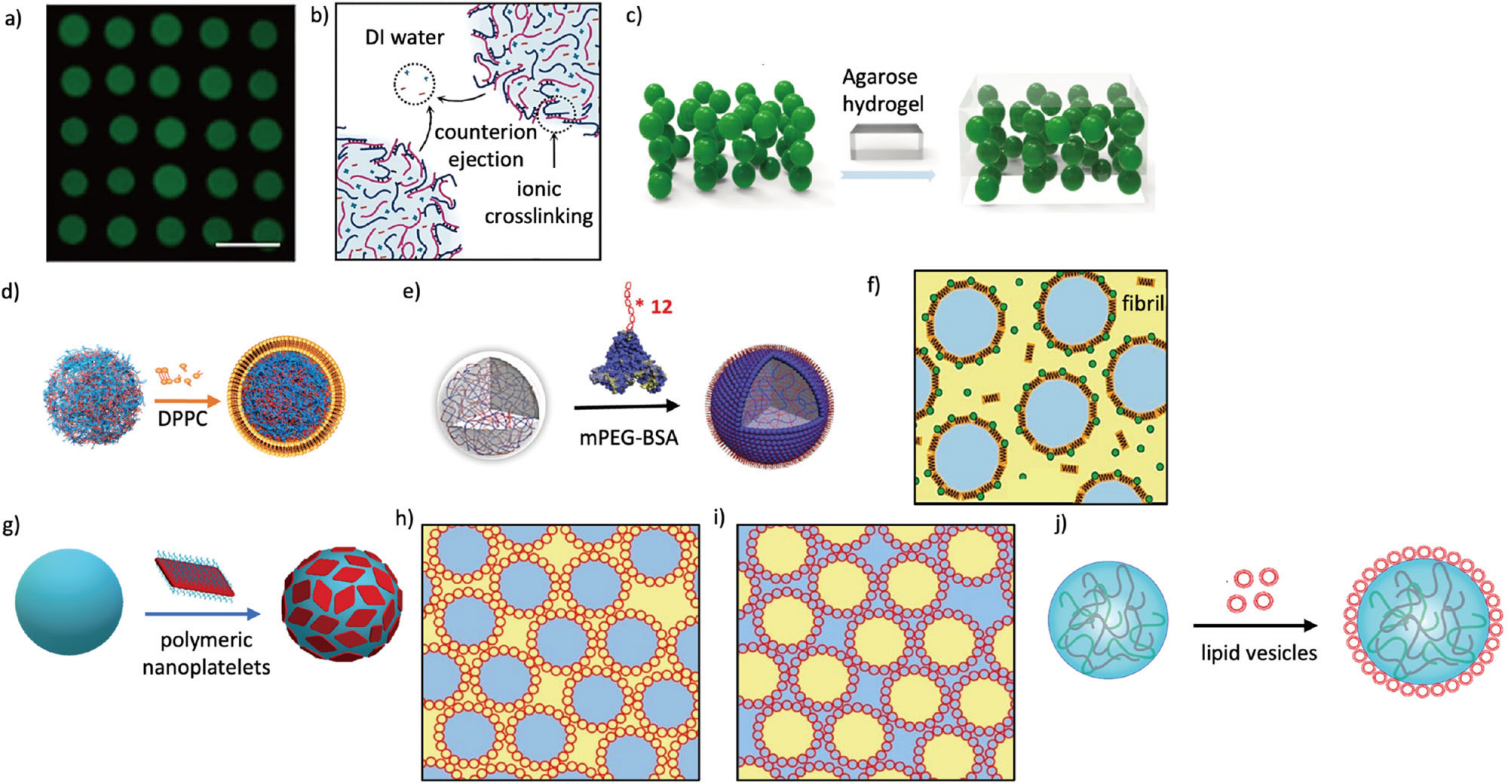
Coacervate stabilization via physical separation methods. **a** Coacervate droplet stabilization via acoustic field implementation (scale bar 150 μm). Adapted with permission from Copyright © 2016, Nature Publishing Group^28^. **b** Coacervate stabilization using electric field showing Illustration of a coacervate droplet interface collapse in DI-water due to ionic crosslinking from interfacial ion ejection. Adapted with permission from Copyright ©2022, National Academy of Science^32^. **c** Matrix-assisted stabilization of coacervate droplets with hydrogel immobilization of coacervate microdroplets. Adapted with permission from Copyright © 2020, Wiley VCH GmbH^33^. **d** Membranization-induced stabilization of LLPS droplets; phospholipid-mediated stabilization of giant coacervate vesicles. Adapted with permission from Copyright © 2021, American Chemical Society^37^. **e** Protein-polymer conjugate membrane-stabilization of coacervates. Adapted with permission from Copyright © 2019, Wiley-VCH GmbH^39^. **f** Protein nanofibril-mediated stabilization of a PEG/Dextran ATPS system. Adapted with permission from Copyright © 2016, Nature Publishing Group^47^. **g** 2D polymer nanoplatelets induced stabilization of PEG/dextran ATPS system. Adapted with permission from Copyright © 2016, American Chemical Society^48^. **h**, **i** Liposome-stabilized PEG-dextran ATPS system (blue, dextran; yellow, PEG). **h** Dextran-rich droplets dispersed in PEG-rich continuous phase, (**i**) PEG-rich droplets dispersed in dextran-rich continuous phase. Adapted with permission from Copyright © 2014, Nature Publishing Group^133^. **j** Lipid vesicle coating to stabilize complex coacervates. Adapted with permission from Copyright © 2019, American Chemical Society^142^.

Acoustic forces, generated by ultrasonic waves, can be used to trap and stabilize LLPS droplets. The pressure variations in the acoustic field create low-pressure region nodes and high-pressure region antinodes^28^. Droplets can be trapped at the nodal regions to prevent coalescence, allowing formation of droplet arrays that respond to chemical gradients and mimic morphogenesis^29,30^ (Fig. 3a). The acoustic wave patterning is also further manipulated for the spatial organization of higher-order droplet colonies as well as their communication with living cells^31^. Mimicking complex patterns in living systems requires further development of more complex droplet patterns under an acoustic field. Although acoustic forces allow for precise spatial arrangement of droplets and the formation of complex patterns, potential drawback of this system is the scale-up challenge; Furthermore, the consistent stability of droplets under constant acoustic pressure is not well-established, which may be a limitation for processes requiring extended periods of stabilization. Moreover, the heat generation through ultrasonic energy could be a concern for temperature-sensitive applications. In another approach, an electric field has been used to manipulate stabilized coacervates^32^. Exposure of polyelectrolyte-based coacervates in deionized water results in stabilized droplets while retaining electrostatic charge and polarizability. Thus, using low-voltage electric fields enables precise positioning of the droplets and formation of chain-like structures (Fig. 3b). Electric fields offer the advantage of rapid and precise droplet manipulation, which is essential for constructing chain-like structures. However, the reliance on aqueous environments with specific ionic strengths could be a hindrance for certain applications. So far, only polyelectrolyte-based coacervates have been employed, although it seems feasible that a range of biomolecule-based coacervates with similar physicochemical properties could be manipulated by using electric field gradients.

Embedding LLPS droplets within a matrix offers an alternative method for droplet stabilization. The matrix acts as a physical barrier, ensuring droplets remain separated and preserving distinct phases^33^ (Fig. 3c). Even though the diffusion of substances may be limited within the matrix, and compatibility between the matrix and droplet contents is required, these initial results offer a step towards diverse applications ranging from controlled drug release, tissue engineering to bioreactors.

In contrast to using matrix to create an integrated 3D environment in LLPS droplets stabilization, membranization as a strategy for stabilizing LLPS droplets provides a physical barrier for individual droplets against droplet fusion^34^. Amphiphilic small molecules can self-assemble into a layer at the interface of LLPS droplets, host phase separation of ATPS^35^, or act as a protective shell to prevent droplet coalescence^36^. In the latter case, fatty acid-coated droplets rapidly respond to changes of ionic strength, allowing for dynamic control over droplet disassembly and membrane fusion. Moreover, higher coacervate loading efficiency, permeability and membrane stability can be achieved by replacing the fatty acid with a phospholipid^37^ (Fig. 3d). These results demonstrate that the property of membranized droplets is determined by delicate interactions between the small molecules and coacervate phase. So far, the use of amphiphilic molecules to create a membranized barrier around droplets can enhance loading efficiency and provide dynamic control over droplet stability. However, the stability of these membranes is highly sensitive to environmental changes such as ionic strength, which could pose challenges in maintaining the desired state of the droplets. Given that the lipid membranes are sensitive to environmental changes, metal-organic systems with enhanced stability have been used to membranize coacervate droplets^38^. Polyphenols coordinate with metal ions can form the metal–phenolic networks (MPNs) that can fast self-assemble from various building blocks on the surface of the coacervates and provide the protocells with physical and chemical stability, surface modifiability and high biocompatibility. Furthermore, MPN membrane have the controlled permeability and it can effectively encapsulate bioactive components such as enzymes and maintain their activities. However, the fixed permeability and membrane stability may limit the application of this system in drug release. Apart from small molecules, polymers including biomacromolecules^39^, polyelectrolytes^39–41^ and synthetic polymers^42^ have also been used in the stabilization of LLPS droplets. For example, membranized structures formed by protein-polymer conjugates can coat single droplets^39^ (Fig. 3e) or trap multiple coacervates^43^. The high permeability of protein-based membranes allows the internalized droplets to sense and process extracellular signals and adapt their behaviors^44^. Fully synthetic polymers such as triblock copolymers with hydrophilic-hydrophobic-hydrophilic sequences have also been utilized to prepare membranized droplets^42,45^. Compared to small molecules, the higher chemical versatility and tunability of block copolymers offer more possibilities to engineer complex interactions. However, the specificity of the interactions these copolymers engage in with the droplet interfaces requires precise molecular engineering, which can significantly increase research and development efforts and costs. Concerns about the biocompatibility and toxicity of synthetic polymers also necessitate rigorous testing, particularly for biomedical applications.

Nano/micro-objects offer a unique approach for droplet stabilization. The earliest example focused on using nanoparticles to stabilize ATPS systems^46^, which inspired intensive investigation of ATPS droplet stabilization with nano-objects such as nanowires^46,47^ (Fig. 3f), nanoplates^48^ (Fig. 3g), nano-microgels^49^ and nanorods^50^. Advances in understanding the interactions between nanoparticles and liquid interfaces has been extended to coacervate systems in which metal nanoparticles were used to stabilize the LLPS droplets^51^. Owing to the responsiveness of nanoparticles by facile surface modification, the disassembly of nanoparticle-jammed membranes can be triggered by external stimulus, resulting in the uptake of guest protocells. Interestingly, nano-objects with hollow structures can also be used for droplet stabilization. For example, nano-sized vesicles have been demonstrated to stabilize ATPSs^51^ (Fig. 3h, i) and coacervates (Fig. 3j). The concentration of vesicles controls the size of the droplets and the loose packing of vesicles on the interface give rise to high permeability. Currently the inner spaces of vesicles are underutilized, although we anticipate that vesicles loaded with functional molecules could lead to materials exchange between the vesicles and droplets and concomitant multi-compartmentalized reaction networks. So far, nanoparticles and micro-objects offer unique possibilities for droplet stabilization, with the added benefit of being able to trigger irreversible disassembly of membrane through external stimulus. We anticipate membranes capable of reversible assemble and disassemble will be developed for higher-level control over the droplet dynamics for complex cytomimetic behaviors.

**Fig. 4:**
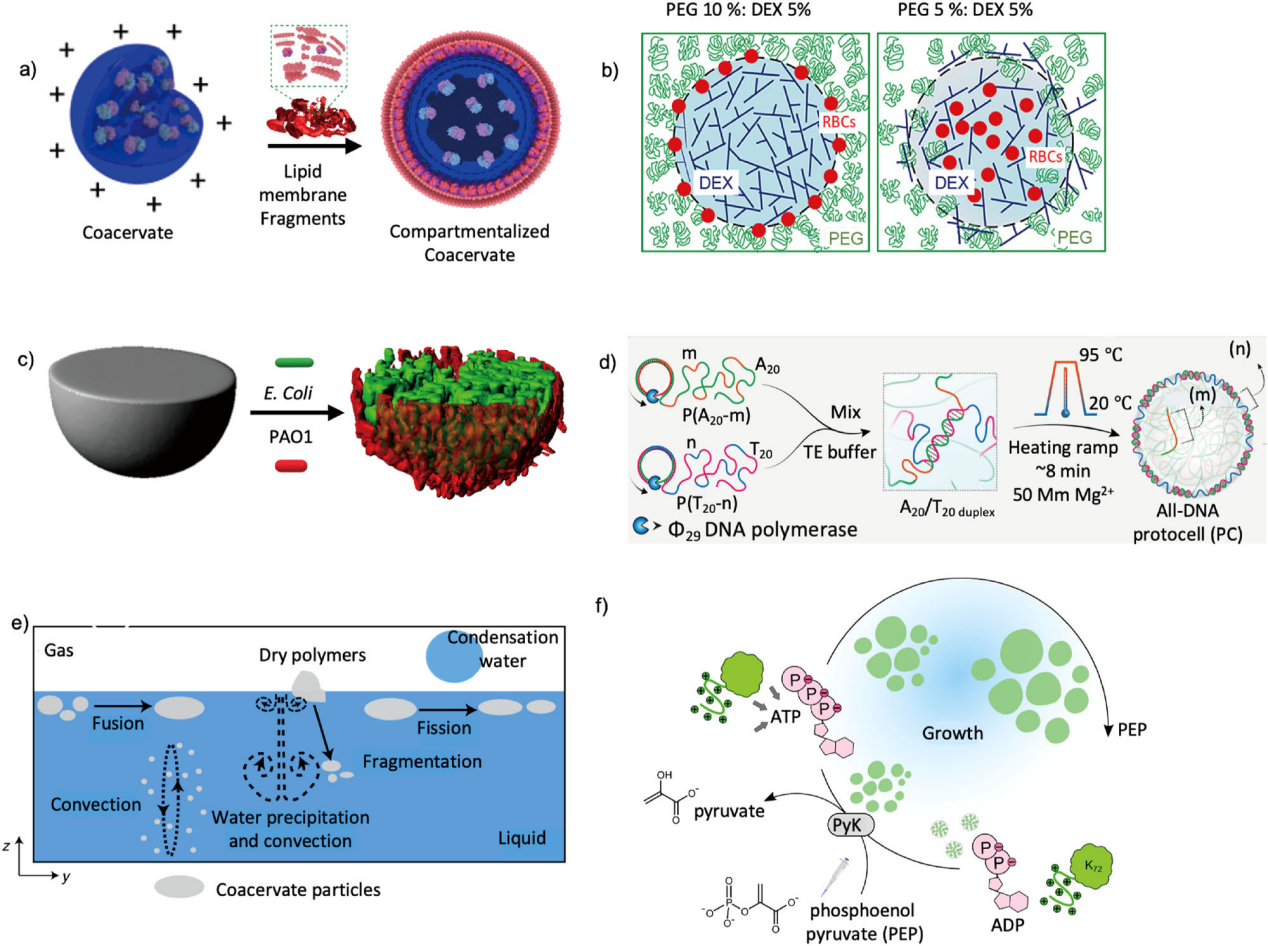
LLPS droplet stabilization using living cell materials, chemical modulation and manipulation of out of equilibrium conditions. **a** Coacervate stabilization using red blood cell (RBC) membrane fragments. Adapted with permission from Copyright © 2020, Nature Publishing Group^53^. **b** PEG/dextran LLPS droplet ATPS stabilization using living cells. Adapted with permission from Copyright © 2019, Frontiers Media S.A^54^. **c** Coacervate formation and stabilization using E. Coli and PA01 bacterial strains. Adapted with permission from Copyright © 2022, Nature Publishing Group^56^. **d** DNA-based protocells composed of dual barcode components with complementary pairs. Adapted with permission from Copyright © 2022, Nature Publishing Group^60^. **e** Coacervate stabilization via maintaining continuous non-equilibrium conditions inside rock pores. Adapted with permission from Copyright © 2022, Nature Publishing Group^63^. **f** Stabilization via continuous chemical fuelling of ATP to the coacervates. Adapted with permission from Copyright © 2021, Nature Publishing Group^21^.

It is also possible to stabilize LLPS droplets through the use of living systems. First attempts have used purified yeast cell fragments^52^ or erythrocyte membrane fragments^53^ (Fig. 4a) to make biologically derived membranes for droplet stabilization. The resulting compartments show remarkable biocompatibility and strong interactions with living cells. However, the potential for immunogenic responses when these biological materials are introduced into new environments or hosts is a significant concern, especially for therapeutic applications, as it could lead to adverse reactions. Furthermore, the complexity of the biological membranes themselves may introduce unpredictability in their interaction with the encapsulated materials or the surrounding medium, potentially affecting the stability and functionality of the stabilized droplets. Alternatively, LLPS droplets can interact directly with living cells to form membranized structures. Droplets of PEG/dextran systems can be stabilized by living cells^54^ (Fig. 4b) and biocompatibility of the ATPS droplets retain cell functions and support cell growth^55^. In contrast, coacervates are prone to rupture living cells, sequester cellular components and extract the lipids for membranization^56^ (Fig. 4c), which is an efficient approach to construct hybrid cells^57^. We expect new coacervate systems will be developed in the future that can be stabilized by intact and functional living cells without killing the cells, offering a wider range of biological applications.

### Chemical modulation

In addition to physically separate droplets and maintain their stabilities, chemical modulation serves as an alternative strategy for stabilizing LLPS droplets which relies on altering the chemical compositions or structures of the droplets. One prevalent method within this domain is the introduction of specific reactive crosslinkers that results in a crosslinked network ensuring the droplets resist coalescence and maintain their distinct structures^58^. Another avenue of crosslinking utilizes non-covalent interactions, providing micro-structures with a liquid core and crosslinked shell^59^ (Table 1). The advantage of these systems is the dynamic nature of the crosslinked shell, giving rise to advanced adaptive behaviors^60,61^ upon stimulus (Fig. 4d). While the aforementioned examples form structures governed by thermodynamic equilibrium or kinetic traps, out-of-equilibrium structures are more ubiquitous in living systems. The concept of out-of-equilibrium involves maintaining a system in a dynamic state through a continuous supply of energy, which otherwise would transform into a state of thermodynamic equilibrium^62^. Physical non-equilibrium conditions have been shown to maintain coacervate sizes reminiscent of Nature^63^ (Fig. 4e). Recently, coacervate systems under out-of-equilibrium conditions have been achieved by chemical fueling, in which ADP as a substrate is converted to ATP by an enzymatic reaction and forms coacervates with a lysine-rich protein (Fig. 4f). Surprisingly, the growing droplets resist Ostwald ripening by both strong electrostatic interactions and entropy effects^21^. Another example includes the active droplets that can undergo a morphological transition into a liquid, spherical shell by continuous supply of high energy molecular fuel^64^. Instead of physical separation such as membranization, these systems provide an appealing chemical method to stabilize droplets against Ostwald ripening.

## Applications of LLPS droplets

In parallel with the development of strategies for droplet stabilization, the unique dynamic features, high tunability and responsiveness of LLPS droplets give rise to functional materials capable of promising applications. In this section, we describe methods employed to regulate the dynamics of LLPS droplets, along with their biomimetic scaffold functions in modeling cellular structures, functions, and higher-order behaviors. Additionally, we describe the biomedical applications of droplets for diagnosis, drug delivery and therapy.

### Biomimetic LLPS droplet formation and dynamics

Compartmentalization is a fundamental characteristic of living systems. To mimic the complex functions of living cells, it is crucial to construct protocells with hierarchical structures that have multiple compartments for organization of materials and biochemical reactions. In addition to membrane-bounded artificial cells and organelles^65,66^, regulation of the formation and dynamics of LLPS droplets provides an alternative strategy of multi-compartmentalization for precise spatiotemporal regulation of reaction networks. In this regard, extensive explorations have been made to control the behaviors of LLPS droplets through physical parameters including pH^67,68^, light^69^, temperature^70^ and ionic strength^71^ and chemical signals^72^. Chemical strategies regulate droplet behaviors with greater diversity, which are discussed below.

**Fig. 5:**
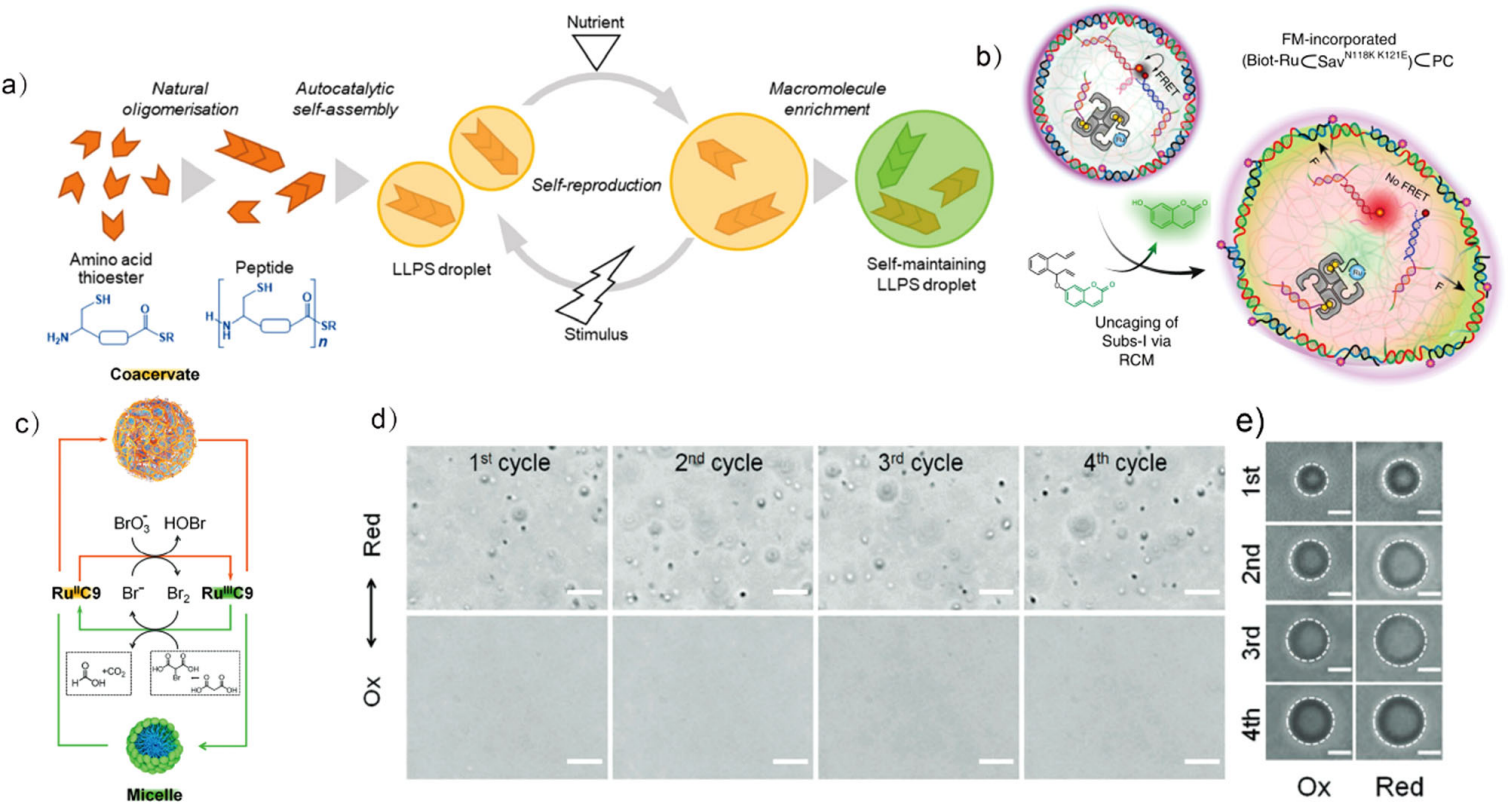
Biomimetic LLPS droplet formation and dynamics. **a** The amino acid thioester is oligomerized to produce a peptide and acts as a source of “nutrition”. A physical stimulus causes droplet division while self-reproduction occurs by incorporation of the nutrients. Adapted with permission from Copyright © 2021, Nature Publishing Group^74^. **b** Immobilized artificial metalloenzymes (ArM) catalyzes an DNA-orthogonal uncaging reaction in DNA protocells (PCs). The uncaged product induces swelling and destabilizes DNA force-sensing modules (installed in the PCs), further triggering the fluorescence output and the membrane dynamization of the protocells. Adapted with permission from Copyright © 2020, Nature Publishing Group^61^. **c** Oscillatory transformation of membraneless microdroplets from LLPS of metallosurfactants (top) and spherical micelles (bottom) by coupling salt-induced coacervation with the BZ reaction in which RuC9 (the metallosurfactant with a ruthenium (II) tris(bipyridine) complex headgroup and two nonyl tails) serves as a catalyst and is repeatedly switched between the oxidized (Ru^III^C9) and reduced states (Ru^II^C9). **d** Optical microscopy images of repeated death/regeneration cycles of droplets; **e** A gradual increase in droplet size is noted at both oxidized and reduced states. Scale bars: (**d**) 5 μm; (**e**) 1 μm. Adapted with permission from Copyright © 2023, Wiley-VCH GmbH^81^.

Increasingly, there is a trend toward developing the active proliferation of LLPS droplets to mimic life cycles of growth, replication and division orchestrated by non-equilibrium complex molecular networks. To achieve this, dissipative conditions have been coupled to coacervation processes enabling droplets that resist Ostwald ripening^21^ and perform “species selection” in an artificial evolutionary scenario^73^. For example, Muneyuki et al. constructed peptide-based droplet protocells that are able to undergo steady growth–division cycles^74^ (Fig. 5a). Oligomerization of amino acid thioester precursors results in formation of peptides and concomitant coacervation that can grow and divide upon periodic supply of precursors and mechanical agitation. Additionally, the protocells actively recruit RNA molecules to protect themselves from dissolution by destabilizing lipids. This system could be further explored by adding mutation and selection mechanisms to achieve Darwinian evolution of the protocells: that is, self-sustained droplets with increased complexity adapt to the environment and eventually obtain new functions^75^.

Besides small molecules, peptides and proteins, DNA may offer alternative strategies to form LLPS droplets by electrostatic interactions^76^ or via programmable base pairing interactions. It has been demonstrated that the dynamic functions of DNA droplets including fusion and fission can be controlled by rational sequence design^8^. Using chemical fueling and enzymatic reactions, DNA-based droplets exhibit transient structures with tunable lifetimes^77^. In another example, Walther et al. introduce abiotic catalytic activities in DNA protocells, allowing the processing of non-DNA signals and giving morphological protocellular changes as response. Artificial metalloenzymes localized inside the DNA droplets activate the precursor via ring-closing metathesis giving rise to a DNA-duplex-weakening product, which can interact with droplet components for dynamic behaviors such as growth, adhesion and fusion^61^ (Fig. 5b). This work demonstrates the potential to build responsive DNA-based droplets that are compatible with various inputs and outputs, such as small molecules^78,79^ and proteins^80^.

Oscillatory phenomena is abundant in biological systems across different scales: from metabolic and biochemical oscillations to embryonic oscillators and circadian clock. A recent development has mimicked oscillating behaviors using coacervates^81^ based on an amphiphilic metal-organic complex that only phase separates when the metal is in a reduced oxidation state (Fig. 5c). Using the Belousov–Zhabotinsky (BZ) reaction to periodically switch the redox states of the metal results in oscillation of the droplets in expansion-contraction-dissociation cycles (Fig. 5d). Due to the transient sequestration ability during oscillation, this work provides opportunities for producing smart microrobots that can periodically release cargos. However, the metal-organic complex functions as both a component of the oscillators and the coacervates, which requires careful optimization. Alternatively, a small molecule oscillator has been demonstrated to catalyze an independent reaction without affecting its oscillating properities^82^, which could minimize the interference between oscillation and coacervation of the aforementioned systems.

In addition to growth, division and dissolution, physical and chemical signals can trigger the transformation of LLPS droplets, such as from a single condensed state into a multiphase^83^, droplets into solid^13^, fibrous^84^, or membranized structures^58^ and liquid crystals^85^, and vesicles into condensates^86^. In general, revealing the mechanism of transformation of LLPS droplets offers not only insights associated with neurodegenerative disease and cancer, but also a method to construct force-responsive materials.

### LLPS droplets as scaffolds in biomimetic cellular systems

**Fig. 6:**
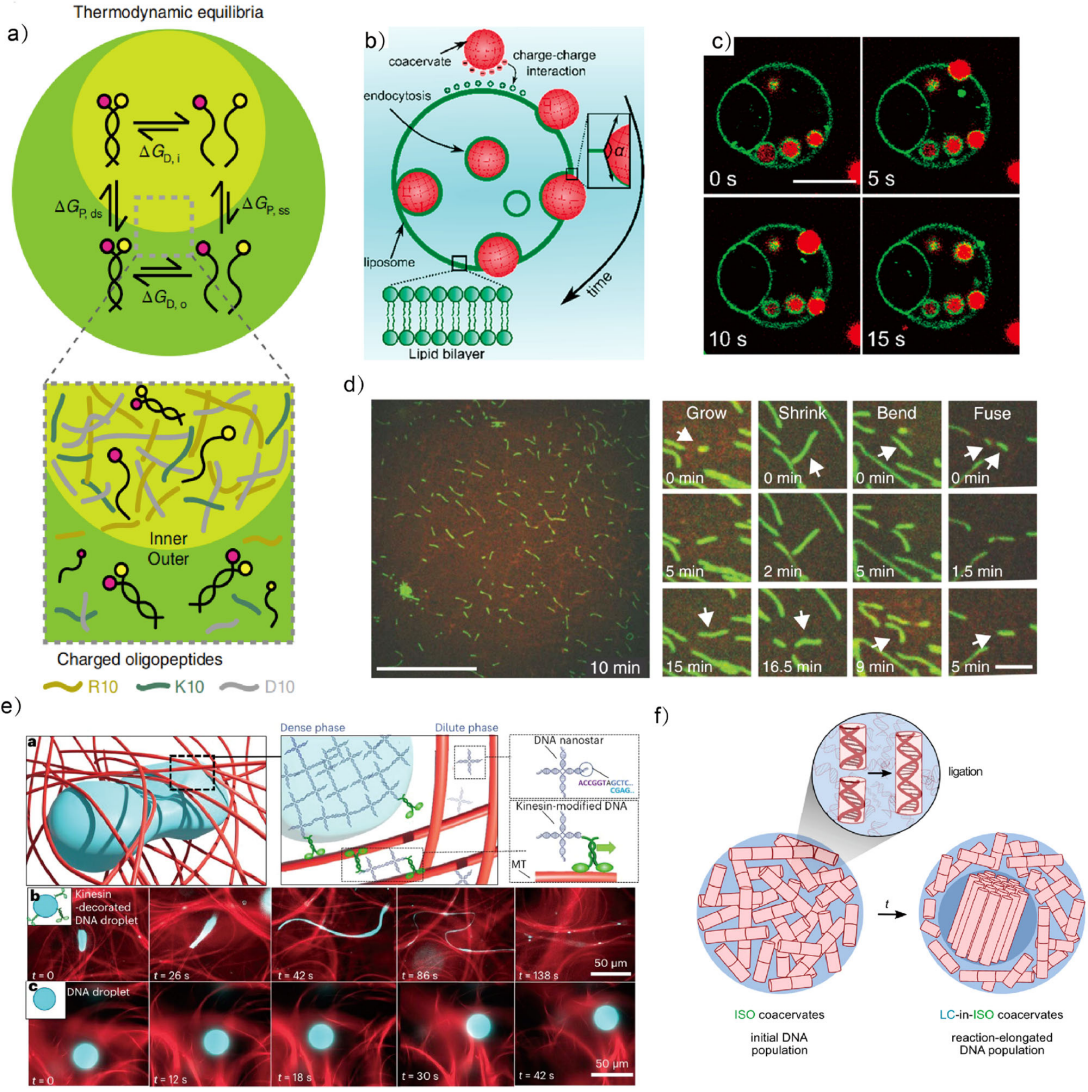
Biomimetic LLPS droplets; multiphase behavior, endocytosis, filament assembly and liquid crystallinity. **a** Single-stranded and double-stranded RNA preferentially accumulate in different phases of the same droplet due to the new thermodynamic equilibrium between RNA partitioning and dissociation introduced by multiphase structures. Adapted with permission from Copyright © 2022, Nature Publishing Group^88^. **b** Schematic illustration of endocytosis of coacervates by liposomes. **c** Time-lapse microscopy of endocytosis after mixing of coacervate droplets with liposomes; Permine/polyU coacervate droplets end up inside the liposomes. Scale bars: 10 μm. Adapted with permission from Copyright © 2022, American Chemical Society^102^. **d** Snapshot of fibrils formed at FtsZ and GTP, showing dynamic behavior, including growth, shrinkage, bending and fusion. Scale bars: 30 μm (large image) and 5 μm (small images). Adapted with permission from Copyright © 2018, Nature Publishing Group^105^. **e** DNA droplet enveloped by an active microtubule (MT) network. Kinesin–DNAs have a motor attached to one arm, whereas the other three arms have attractive interactions. They bond droplets to MTs, and generate autonomous flows in the dilute phase. Adapted with permission from Copyright © 2023, Nature Publishing Group^107^ **f** Structural transformation of the coacervate droplets into multiphase LC-in-Isotropic (ISO) coacervate droplets during DNA Dickerson dodecamer sequence (DD) oligonucleotide ligation. Adapted with permission from Copyright © 2023, Nature Publishing Group^85^.

Biomolecular condensates in eukaryotic cells control diverse biological functions by organizing complex biochemical reactions in space and time^1^. To understand the underlying physicochemical principles of biomolecular condensates and use them to design advanced functions for synthetic biology, LLPS has been incorporated into artificial cellular systems as scaffolds for housing endogenous processes. The partitioning of client molecules in LLPS droplets is determined by multiple factors including the porosity of the droplet, and non-specific and specific interactions^2,87^. Inspired by the multiphase structures in living cells, a recent study has demonstrated that RNA partitioning and duplex dissociation are phase-specific in synthetic multiphase droplets^88^ (Fig. 6a). Single-stranded and double-stranded RNA preferentially accumulate in different phases of the same droplet due to the thermodynamic equilibrium between RNA partitioning and dissociation within the multiphase structures. This research elucidates the mechanisms of molecular partitioning and reaction equilibrium of condensates in living cells and lays the foundation for the construction of functional artificial cells with multiphases capable of performing multiple reactions separately and simultaneously.

Partitioning of client molecules into the droplets confers diverse functions. By recruiting nucleic acid as clients, droplets support RNA copying, RNA aptamer binding and catalytic functions of DNAzyme and RNAzyme^89,90^. Interestingly, coacervates made from RNAzyme and peptide show catalytic properties without addition of a client with enzymatic activity^91^. Consequently, the RNAzyme functions as a scaffold while sustaining the ribozyme activity, suggesting an alternative method to construct catalytically active droplets that would otherwise require additional functional clients^92^.

Along with catalytic nucleic acids, proteins can also maintain their enzymatic activity in LLPS droplets for small molecule conversion^93^ and in vitro transcription/translation^94^. Additionally, enzymatic reactions can induce inherent flow^95^ and motion of droplets^96^. In the latter case, coacervates are coated with an enzyme-conjugated polymer membrane. The fluidity of the polymer membrane causes a transitory asymmetric distribution of the propulsive units (enzymes), leading to macroscopic movement of the coacervate droplets. The results demonstrate that stochasticity could be a useful mechanism to construct adaptive droplet protocells featuring chemotaxis^97^.

A particularly promising class of droplets are all-DNA coacervates^59^, since they offer selective client molecule uptake based on base-pairing interaction. It has been demonstrated that DNA droplets function as a scaffold for DNA computing and reaction-diffusion patterning^98^. We anticipate that DNA droplets could serve as scaffolds to selectively enrich various functional molecules such as enzymes and nanoparticles and perform highly parallel chemical reactions that match the complexity in living cells.

### Interaction of LLPS droplets with biomimetic membranes and cytoskeletal scaffolds

The interaction between biological condensates and membranes is of great significance in cell signaling^99^ and cell adhesion^100^. To reveal wetting-mediated interactions between membranes and droplets from a bottom-up perspective, LLPS systems have been mixed with liposomes to observe how different wetting morphologies are governed by adhesion, interfacial tension and membrane elasticity^101^. In other studies, interactions between coacervates and liposomes lead to engulfment of the droplets^102^. (Fig. 6b), suggesting a new direction for intracellular delivery although exocytosis of the coacervates from the liposomes remains a current challenge (Fig. 6c). In contrast to directly mixing liposomes with pre-existing LLPS droplets, in situ formation of droplets within liposomes results in phase separation at the membrane boundary and membrane budding, which together provides a method to construct multi-compartments within artificial cells^103^.

Biomolecular condensates and cytoskeletal filaments regulate each other in living cells^104^. Inspired by nature, specialized proteins have been used to investigate the interactions between coacervates and protein filaments. For example, dissipative self-assembly of FtsZ (a bacterial homolog of tubulin) has been achieved within coacervate droplets^105^ (Fig. 6d). Adding guanosine triphosphate as fuel causes localized fibrous elongation and division. Fibrous structures can also affect the dynamics of droplets from outside^106^. A recent development uses microtubule-based dynamic networks to regulate the phase separation of DNA-based droplets (Fig. 6e). The modification of kinesin molecular motor on the DNA droplets is identified as key factor, as it establishes the mechanical bond between the droplets and filaments. This work provides a new platform for simulating collaborative motions of fibers and droplets in artificial cells and demonstrates the potential for applications as active soft materials. In contrast to specialized proteins, synthetic peptides have been reported to form fiber-containing liquid droplets^107^. We envision more fully synthetic materials will be used to develop minimalistic multicomponent systems featuring co-existence of fibers and droplets. Finally, as current investigations largely focus on the interactions between LLPS with either membranes or filaments, the next key step is to implement the integration of membranes, fibers and LLPS to produce LLPS-based artificial cells with multiple functions^108,109^.

### Liquid–liquid crystalline phase separation

LLPS and liquid crystalline (LC) ordering occur together in Nature; however, we are only beginning to understand the connections between LLPS and LC ordering and use synthetic systems to mimic these processes^110^. In an illustrative example, self-assembly of cellulose nanocrystals in PEG/dextran two phases system leads to multiphase separation, generating overlapping behaviors of LLPS and LC termed liquid–liquid crystal phase separation (LLCPS)^111^. Excitingly, it has been shown that LCs give rise to enhanced functionality of LLPS droplets^85^ (Fig. 6f). The rate of oligonucleotide ligation is increased within LLCPS systems containing azobenzene cations and DNA stacking, where the LC phase facilitates organization of the reactive oligonucleotides and the fluidity of the system allows for efficient materials transportation. This work should stimulate further investigations on LLCPS with diverse catalytic activities.

### LLPS droplet-based biomimetic communication

**Fig. 7:**
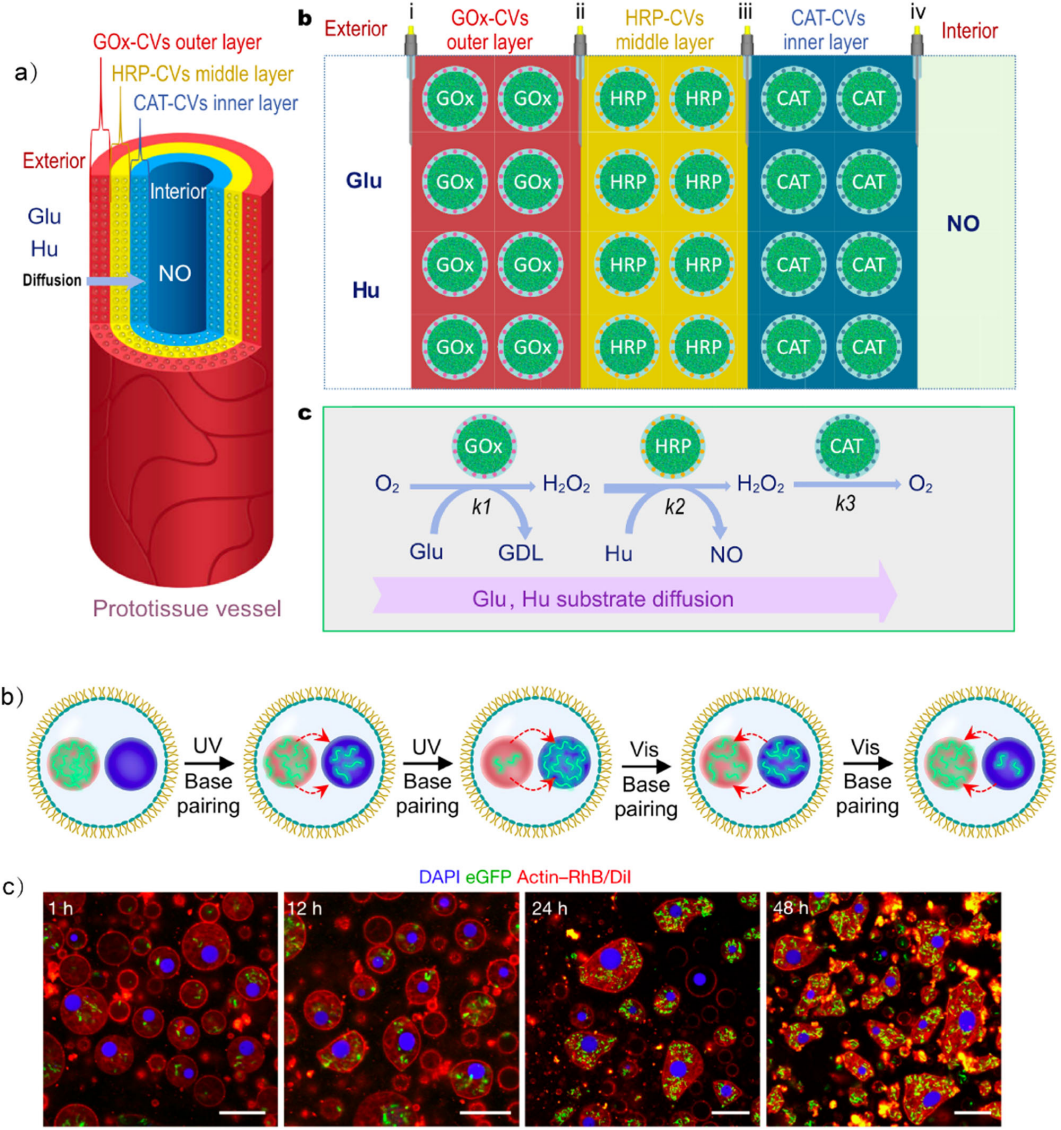
LLPS droplet-based biomimetic communication. **a** The tubular three-layer model prototissue vessel and the communication pathways between LLPS protocells. It immobilized populations of GOx-CVs, HRP-CVs or CAT-CVs in the outer, middle or inner hydrogel modules. Enzyme-decorated coacervate artificial cells process multiple signaling molecules involved in an enzyme cascade reaction. Adapted with permission from Copyright © 2022, Nature Publishing Group^113^. **b** Communication between LLPS droplets as artificial organelles. Schematic drawings and confocal images showing the exchange of FITC-DEAE-Dex between two DNA coacervates (labeled by AF405 andCy5). Adapted with permission from Copyright © 2022, Wiley-VCH GmbH^116^. **c** Communication between LLPS droplets and living cells. The living cell-containing coacervate droplets are dynamic in terms of living *E.coli* and F-actin; confocal microscopy images show the morphology transformation from spherical to non-spherical bacteriogenic protocells. Red, F-actin and outer membrane; blue, DNA–histone condensate; green, guest live E. coli cells. Scale bars: 10 μm. Adapted with permission from Copyright © 2022, Nature Publishing Group^56^.

Multicellular organisms composed of various cell types communicate chemically and constantly with one another to coordinate their activities. By taking advantage of the spontaneous sequestration of LLPS droplets, contact-based^112^ and diffusion-based^113^ (Fig. 7a) communication have been established between populations of artificial cells. The ability of coacervates to selectively uptake and release chemical signals is enhanced by the implementation of specific interactions^114,115^, paving the way for the development of bidirectional communication between LLPS droplets^116^ (Fig. 7b).

Another direction of future development concerns the use of LLPS to interact with biological systems^57,117^. The biocompatibility of dextran/PEG two phase systems makes it an excellent platform to interface with living cells^118,119^. A recent development uses photosynthetic algal cell-containing ATPS droplets with a shell of respiratory bacterial cells as bioreactors^120^. The hypoxic photosynthesis and aerobic respiration act synergistically boosting hydrogen generation. Besides segregative phase separation, associative phase separation also has been used to integrate living cells, giving rise to living materials^56^ (Fig. 7c). Rupture of the captured bacterial cells and sequestration of the functional bacteriogenic components, allows the construction of cytomimetic structures comprising a nucleus, vacuoles and cytoskeleton. Subsequently, living cells are introduced into the bacteriogenic protocells as artificial mitochondria to support various enzymatic reactions and protocellular growth. Although these cellular bionic systems cannot match the sophistication of their biological counterparts, the construction of bacteriogenic protocells blurs the boundaries between living and non-living matter and demonstrates a new strategy for engineering living materials^121^.

### Biomedical applications

Liquid droplets formed by LLPS and composed of natural or biocompatible materials enable dynamic performance and can respond intelligently to environmental stimuli, providing opportunities to design smart materials for biomedical applications including diagnosis, delivery and therapeutics.

**Fig. 8:**
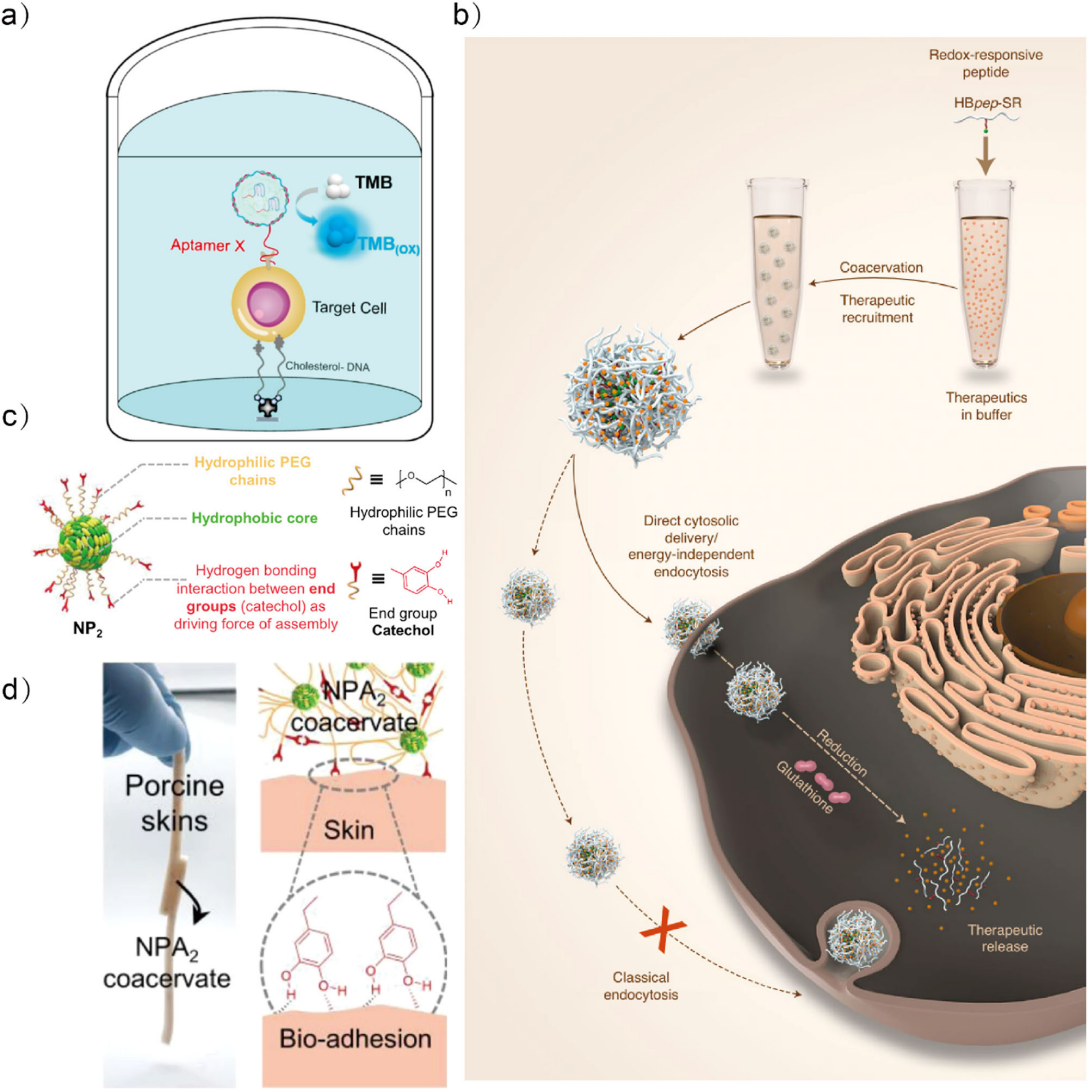
Biomedical applications of LLPS droplets. **a** Schematic illustration of the mechanism of the designed sandwich assay for distinguishing different cancer cells based on aptamer X (in this case, Sgc8 aptamer)-functionalized DNA-encoded artificial cells. Adapted with permission from Copyright © 2023, Wiley-VCH GmbH^122^. **b** Schematic illustration of redox-responsive peptide coacervates hBpep-Sr with direct cytosolic entry that bypasses classical endocytosis. Adapted with permission from Copyright © 2022, Nature Publishing Group^126^. **c** The NPA coacervate contains a hydrophobic core, hydrophilic PEG chains as the shell, and end groups of the PEG chains provide the driving force for self-assembly. **d** NPA coacervate has the bio-adhesion function and it can glue porcine skin tissue together. Adapted with permission from Copyright © 2021, Nature Publishing Group^127^.

LLPS droplets are garnering interests in diagnostic research, as they can selectively recruit biomarkers such as proteins, nucleic acids, and metabolites via noncovalent interactions. For example, localization of a hemin/DNA G-quadruplex complex inside DNA droplets boosts the peroxidase-like activity^122^ (Fig. 8a). The biosensing droplets are designed to detect cancer biomarkers (microRNAs) down to picomolar concentrations and distinguish cancer cells with different membrane protein profiles. In a related approach, DNA coacervates have been used to detect microRNAs with droplet division as readout^123^. Since DNA is scalable, the system can recognize up to four microRNAs simultaneously through computational operations. The detection limit of these systems can be improved by a pre-amplification process. Collectively, these designs offer new insights on the construction of next generation artificial cell-based biosensors.

Coacervates have the benefit of being generated from aqueous solutions, as opposed to many nanoparticle delivery techniques, which frequently require organic solvents. Similarly, biomolecule-based coacervates are relatively safe and easy to manage, allowing them to be manufactured commercially and steadily in the biomedical area. In addition, the responsiveness of the droplets enables targeted release of the drug, thus improving drug utilization^124,125^. Yue et al. created micrometer-sized peptide coacervates as delivery vesicles that can pass the cell membrane without being endocytosed^126^ (Fig. 8b). The pH- and redox-responsive coacervate microdroplets are disassembled in the intracellular environment, thereby efficiently releasing the cargos. Interestingly, the coacervates also prevent RNase-induced premature mRNA cargo degradation, probably because RNase cannot diffuse through the microdroplets after it has formed. In contrast to the conventional notion that delivery vesicles need to be smaller than 200 nm to cross the cell membrane, this study achieves direct cytosolic delivery with micrometer-sized coacervates. Further studies could modify the coacervates with aptamers or antibodies to enhance the target specificity.

Additionally, the versatility and tunability of coacervate-based materials makes them compatible with a wide range of biological diseases. For example, coacervates with wet bioadhesive capability (Fig. 8c, d) have been developed to extend the retention of water-soluble small molecule drugs in the harsh gastrointestinal environment^127^. Owing to the stabilized effect of the nanoparticle assembly, the coacervates are protective against in vivo environmental damage such as extreme pH and salt concentrations. In another example, membranized coacervates capable of producing nitric oxide enzymatically have been exploited for in vitro and in vivo blood vessel vasodilation^53^. Notably, the membranous structures significantly prolong blood circulation time and improve haemocompatibility by preventing direct contact with the living cells, highlighting the potentials of membranized LLPS droplets for in vivo applications.

## Outlook

Although rapid progress has been made in the development of stabilized LLPS droplets as well as their applications in bottom-up synthetic biology and biotechnology, some open challenges remain to be addressed in future work.

The physical properties of LLPS droplets provide valuable guidelines for the construction and modulation strategies employed in various applications. Although the macroscopic physical properties such as viscosity and surface tension have been studied extensively^128^, droplet dynamics at the molecular scale is largely unexplored. In this regard, a combination of spectroscopy and simulations have been used to study protein condensates to gain a comprehensive picture of droplet dynamics^129^, paving the way to the investigation of droplets made from various materials. In addition, although droplet reconfiguration and energy dissipation have been achieved, how these transient structures influence functionally related physicochemical properties remains to be explored.

While membranization effectively provides an enclosing environment to stabilize LLPS droplets, it has yet to be widely used to bring new functions to the droplets. Furthermore, membranization could limit the inherently capability of the droplets to sequester certain biomolecules unless circumvented^41,130^. In this regard, membranized strategies have been demonstrated to determine the permeability of the stabilized droplets^37^, including mechanisms that can dynamically control the permeability^131^.

Living tissues comprise spatially interconnected individual cells that can communicate and behave collectively. Using synthetic cells to build artificial tissues is a major challenge that has important implications in bottom-up synthetic biology and bioengineering of smart micro-devices. Early progress has already been made to use stabilized LLPS droplets to fabricate higher order assemblies^60,132^, although engineering populations of LLPS droplets with orderly organizations^133,134^ and collective behaviors^135^ remains challenging. Promising approaches to precisely arrange stabilized droplets include external controlling techniques including 3D printing^130,136,137^, gel treatment^137^, optical tweezers^138^, and magnetic^139^ and acoustic^30^ fields. These techniques are also compatible with living cells, offering a means to develop artificial cell/living cell hybrid systems. Alternatively, we envision that droplet-based artificial tissues with coordinated functions can emerge autonomously by using chemical reaction networks to control the inter-protocellular interactions. So far, only relatively simple communication pathways have been employed between LLPS droplets, and we anticipate that more complex reaction networks^21,140,141^ will be implemented in the future to construct artificial tissues capable of assembly and function in response to molecular cues. Moreover, opportunities for interfacing droplet-based artificial tissues with living systems based on molecular cues secreted by living cells should also become possible as the communication strategies become more advanced. Ultimately, with LLPS droplets as a class of artificial cells, we expect that stabilized strategies could be crucial for higher-order assembly into artificial tissues, offering significant potential for building responsive life-like multicellular entities for synthetic biology and bioengineering.
